# Case Report: Stepwise quantitative assessment of intraoperative intracranial pressure after perforation, craniotomy, dural incision, and hematoma removal in acute subdural hematoma

**DOI:** 10.3389/fsurg.2026.1862269

**Published:** 2026-06-23

**Authors:** Sota Wakahara, Ryota Tamura, Takahiro Iinuma, Konosuke Ishikawa, Kosuke Karatsu, Ryo Yamamoto, Yukio Sato, Junichi Sasaki, Masahiro Toda

**Affiliations:** 1 Department of Neurosurgery, Keio University School of Medicine, Shinjuku, Tokyo, Japan; 2 Department of Emergency and Critical Care Medicine, Keio University School of Medicine, Shinjuku, Tokyo, Japan

**Keywords:** acute subdural hematoma, craniotomy, decompression, intracranial pressure, intracranial pressure monitoring

## Abstract

**Introduction:**

Severe traumatic brain injury often requires emergency surgical intervention such as craniotomy or decompressive craniectomy. The timely reduction of intracranial pressure (ICP) is critical for improving neurological outcomes. However, limited quantitative data exist regarding how each surgical step of perforation, craniotomy, dural incision, and hematoma removal contributes to ICP reduction in acute subdural hematoma.

**Case presentation:**

A man in his 40s developed bilateral acute subdural hematomas (right/left: 8/7 mm thick) following a fall. His initial Glasgow Coma Scale score was 3. Because the bilateral hematomas were relatively thin, we initially opted for medical management, inserting an ICP sensor into the right frontal lobe for continuous monitoring. Despite initial neurocritical care, ICP remained elevated (45 mmHg). Serial imaging revealed the progression of cerebral edema and midline shift. Emergent decompressive craniectomy on the left side was therefore performed under continuous ICP monitoring. ICP was recorded at each surgical step: single burr hole placement (42 mmHg), partial craniotomy (39 mmHg), complete craniotomy (20 mmHg), dural incision (14 mmHg), and hematoma evacuation (9 mmHg). Each step produced a measurable decrease in ICP. Despite aggressive management, the patient's condition deteriorated; he died on hospital day 21.

**Conclusion:**

The present case illustrates the stepwise contribution of each surgical intervention to ICP reduction in acute subdural hematoma. Although the clinical outcome was unfavorable, these quantitative data provide valuable insights into the decompressive effect of each surgical step. These findings underscore the importance of prompt and progressive surgical management of severe traumatic brain injury with elevated ICP.

## Introduction

Severe traumatic brain injury (TBI) is an urgent condition that often requires emergency craniotomy or decompressive craniectomy. Surgical intervention for severe TBI must be performed promptly, and earlier emergent operations are reportedly associated with better patient outcomes (1). In particular, the mortality rate of acute subdural hematoma (ASDH) has been reported to be as high as 65% when surgical decompression is performed more than 2 h after coma onset, versus just 17% when performed within 2 h after coma onset; these results underscore the critical importance of timely decompression (2).

In emergency departments that manage patients with severe TBI, multiple critically ill patients are often treated simultaneously, which can result in delays before entering the operating room for emergent surgery. In such cases, burr hole surgery may be performed in the emergency department to achieve decompression as promptly as possible (3). There have been reports of the effectiveness of emergency burr hole drainage even in cases of ASDH with bilateral non-reactive pupils, provided that pupil diameter is 4 mm or less on at least one side (4); such clinical effectiveness is frequently observed in practice. However, in clinical practice, there is considerable variation among institutions regarding the implementation of emergency burr hole drainage. Some centers perform it proactively, whereas others do not. Although guidelines exist, institutional policies regarding whether to proceed with aggressive craniotomy also differ, particularly in borderline cases in which decisions rely heavily on clinical judgment. One possible reason for this variability is the lack of quantitative data indicating how much each surgical step—such as placing a single burr hole, performing a craniotomy, incising the dura, and hematoma removal—contributes to intracranial pressure (ICP) reduction.

In the current report, we present a case of severe TBI caused by ASDH, in which decompressive craniectomy was performed under ICP monitoring. This case allowed for the quantitative assessment of ICP reduction at each surgical procedure, including the placement of a single burr hole, partial craniotomy (half of the planned area), full craniotomy, dural incision, and hematoma removal. Herein, we report this case and discuss the effect of each surgical intervention on ICP.

## Case description

A male patient in his 40s with no relevant medical history, medications, or allergies was witnessed falling down 11 steps at a train station, prompting an emergency call. Upon the arrival of emergency medical services, he was found unresponsive. His vital signs were a Japan Coma Scale of III-300, Glasgow Coma Scale of 3, respiratory rate of 30 breaths per minute, heart rate of 108 bpm, blood pressure of 240/106 mmHg, and oxygen saturation (SpO2) of 96% with a reservoir mask at 10 L/minute of oxygen. He also had anisocoria, with his right pupil measuring 4.0 mm and his left pupil measuring 3.0 mm; both exhibited sluggish light reflexes. It was not possible to measure body temperature at the scene. A subcutaneous hematoma of approximately 5 cm in diameter was observed in the right occipital region, along with bleeding from the right ear.

The patient arrived at the hospital approximately 40 min after the injury. On arrival, his consciousness remained impaired with a Glasgow Coma Scale of 3 (E1V1M1). Pupillary examination revealed a right pupil of 3.0 mm and a left pupil of 3.5 mm, both showing diminished light reflexes. To protect his airway, oral intubation was performed and mechanical ventilation was initiated. Non-contrast and contrast-enhanced computed tomography (CT) of the head and torso revealed bilateral ASDH (8 mm thick on the right and 7 mm thick on the left), traumatic subarachnoid hemorrhage, and a right temporal bone fracture (Figure 1A). External examination demonstrated a subcutaneous hematoma extending from the right temporal to the parietal region; no other obvious trauma was identified outside the head.

**Figure 1:**
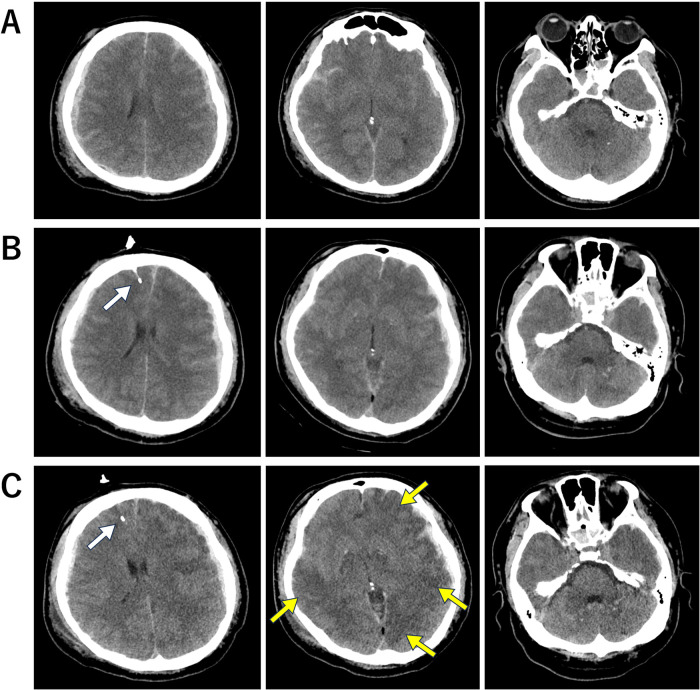
Head CT scans at arrival, 3 h and 12 h after injury. **(A)** Head CT showing bilateral acute subdural hematomas and traumatic subarachnoid hemorrhage at arrival. **(B)** Head CT obtained 3 h after injury, showing persistent bilateral subdural hematomas without significant interval change. **(C)** Head CT showing newly apparent cerebral contusions (yellow arrow) in addition to the previous findings at 12 h after injury. White arrow: ICP sensor.

## Diagnostic assessment

No signs of brain herniation were observed at this time, and a conservative approach without craniotomy was initially chosen. However, given the presence of bilateral lesions and the need for close monitoring, an ICP sensor was inserted into the right frontal lobe and continuous ICP monitoring was initiated. Approximately 1 h after arrival, a Codman® microsensor skull bolt kit (Integra Japan, Tokyo, Japan) was placed, and ICP monitoring was initiated. Although the ICP at the time of sensor placement was 60 mmHg, no signs of brain herniation were present on imaging, and immediate surgical decompression was not performed. Despite the patient's relatively young age, GCS score of 3, and anisocoria, the initial CT showed relatively thin bilateral ASDHs without definite radiological brain herniation or brainstem compression. Given the bilateral nature of the hematomas and the lack of clear radiological indication for immediate bilateral decompression, immediate bilateral decompressive surgery was considered highly invasive and was not selected as the initial strategy. Neurocritical care was initiated while closely observing temporal changes in ICP, and repeated CT findings were used to reassess the need for surgery dynamically. Nevertheless, the initial ICP value of 60 mmHg represented severe intracranial hypertension, and in retrospect, earlier decompressive surgery could have been considered once sustained ICP elevation was confirmed.

Approximately 3 h after the injury, no worsening of the pupillary findings was observed. However, the ICP remained somewhat elevated at 40 mmHg, prompting the intensification of sedation and analgesia. A follow-up head CT at approximately 3 h post-injury showed slight effacement of the parietal sulcus (Figure 1B). Nevertheless, no obvious compression of the brainstem was observed, and neurocritical care was continued under ICP monitoring. This included sedation with midazolam and propofol, analgesia with fentanyl, early post-traumatic seizure prophylaxis with levetiracetam, mechanical ventilator management to maintain partial pressure of CO_2_ (PaCO_2_) between 35 and 38 mmHg, intermittent bolus administration of mannitol, administration of 3% sodium chloride to prevent hyponatremia, target temperature management using an intravascular cooling catheter for neuroprotection with a target temperature of 35 °C, and barbiturate coma therapy. Through neurocritical care, the ICP was temporarily controlled and maintained in the 20–29 mmHg range, although it subsequently increased to 30 mmHg. At this time, the bilateral pupils were 2.5 mm. A repeat head CT at 12 h after the injury revealed newly developed low-density areas in both temporal lobes and the left frontoparietal region, indicating the progression of diffuse cerebral swelling. There were also signs of brainstem compression caused by the narrowing of the basal cisterns, as well as a 5-mm rightward midline shift (Figure 1C).

## Therapeutic intervention

Approximately 10 h after the initial ICP elevation to 60 mmHg at sensor placement, emergent left-sided decompressive craniectomy was performed because the left dominant hemisphere exhibited more pronounced cerebral swelling. The procedure was performed under ICP monitoring, and sequential decreases in ICP were observed during each stage of the surgery. Immediately before the surgery, the ICP was 45 mmHg. After the placement of a single burr hole, the ICP decreased to 42 mmHg (6.7% reduction from the preoperative value). A large frontotemporal craniotomy was planned for decompression; however, because there was strong adhesion between the skull and dura mater, the bone flap had to be divided into two pieces, and the craniotomy was performed in two stages. The ICP was 39 mmHg after the completion of half of the planned craniotomy (13.3% reduction from the preoperative value), 20 mmHg after the completion of the full craniotomy (55.6% reduction), 14 mmHg after the dural incision (68.9% reduction), and 9 mmHg following the evacuation of the subdural hematoma (80.0% reduction) (Figures 2A,B). The final craniotomy measured approximately 11 × 13 cm. To achieve maximal decompression, duraplasty was performed using Durabeam® (Tama Bio, Tokyo, Japan), which was sutured to the native dura mater. The bone flap was not replaced for external decompression. By the end of the operation, the ICP had decreased to 10 mmHg.

**Figure 2:**
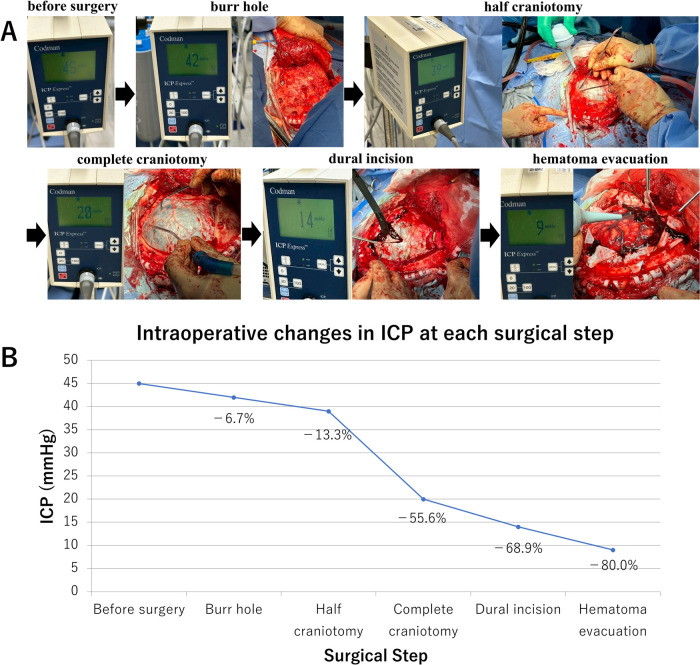
Intraoperative changes in intracranial pressure (ICP) at each surgical step. **(A)** Intra-operative photographs of each step and the corresponding ICP. (The photo of burr hole shows the operative view after additional burr holes were created). **(B)** Graph of ICP changes.

## Follow-up and outcomes

Postoperative CT confirmed adequate decompression and the improvement of brainstem compression; however, newly apparent cerebral contusions and the expansion of cortical low-density areas were observed (Figure 3A). Postoperatively, the ICP remained between 10 and 15 mmHg; however, approximately 5 h after surgery, it increased again to over 30 mmHg. A head CT scan obtained at the time of ICP elevation revealed the further progression of cerebral contusions and the expansion of low-density areas, suggesting the development of delayed brain injury caused by elevated ICP (Figure 3B). Given that improved neurological outcomes through additional invasive procedures were not expected, and that the patient's family declined further surgical intervention, it was decided to continue with medical management only. On hospital day 4, the patient's ICP increased to 40 mmHg, and follow-up CT imaging showed worsening findings (Figure 3C). His systemic condition gradually deteriorated, and he died on hospital day 21.

**Figure 3:**
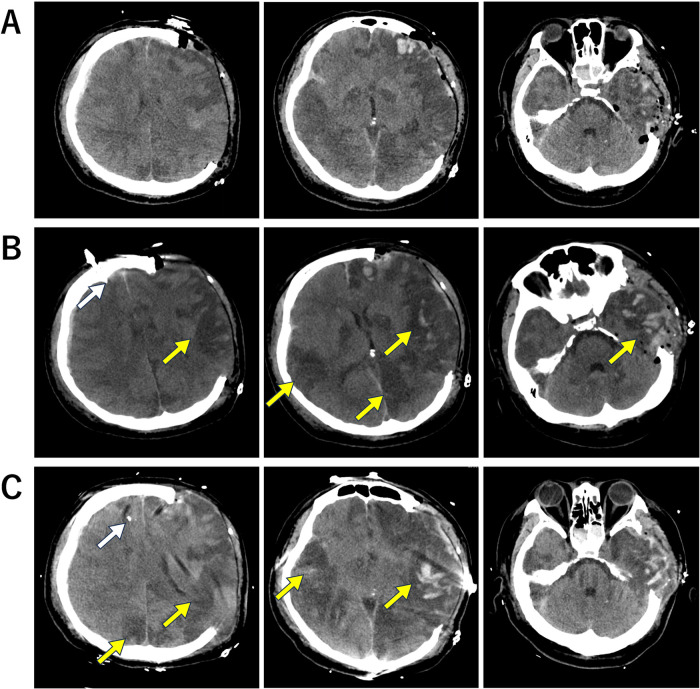
Head CT scans after decompressive craniectomy. **(A)** Head CT immediately after decompressive craniectomy for hematoma evacuation. **(B)** Head CT showing the emergence of cerebral contusions and expansion of cortical low-density areas (yellow arrow) at 5 h postoperatively. **(C)** Head CT demonstrating worsening of the aforementioned findings (yellow arrow) at 4 days after injury. White arrow: ICP sensor.

## Discussion

In the present case of severe TBI caused by ASDH, neurocritical care was provided in accordance with the Seattle International Severe Traumatic Brain Injury Consensus Conference (SIBICC) guidelines (5). Despite both medical and surgical interventions, the patient developed progressive secondary brain injury as a result of sustained intracranial hypertension and ultimately died. Although a favorable outcome was not achieved, this case allowed for a quantitative assessment of the stage-specific reduction in ICP that is achieved through surgical intervention.

Although reports indicate that burr hole surgery for ASDH can reduce ICP (with measured values) (6), and that ICP changes from the preoperative state through craniectomy and dural incision have also been quantified (7, 8), such reports remain limited. The data in the previous reports showed considerable variability, and because the present report also represents only a single case, it remains difficult to generalize how much ICP may decrease following each surgical step. Nonetheless, the relevant findings are consistent with the underlying pathophysiology, and many emergency physicians and neurosurgeons have likely encountered cases in which anisocoria improved following this procedure in the emergency department.

In the initial management of severe TBI, emergency departments are often in a state of urgency because there is a need to address multiple systemic issues, including airway management and the correction of coagulopathy. Moreover, facilities that accept patients with severe TBI often concurrently manage other critically ill patients who require surgical treatment, and the timely transfer to an operating room may not always be feasible. In such situations, mannitol is frequently administered because it improves pupillary abnormalities and neurological outcomes, and may reduce ICP (9). However, it has been reported that single burr hole surgery may lead to better neurological outcomes than mannitol administration as a bridge to craniotomy (10). At our institution, we actively perform burr hole drainage in the emergency department as a relatively quick and streamlined procedure.

In the present case, the ICP decreased in proportion to the extent of the craniotomy, and reductions were observed at each subsequent step, including the dural incision and evacuation of the subdural hematoma. Although this concept is theoretically well understood, we believe that this case is valuable to quantitatively visualize and confirm a reduction in ICP at each surgical step. Notably, doubling the extent of the craniotomy alone contributed to a marked decrease in ICP, from 39 to 20 mmHg. In decompressive craniectomy, a craniotomy size ≥ 12 × 15 cm or a diameter ≥ 15 cm is recommended (11). Additionally, it has been reported that a maximum anterior–posterior craniectomy diameter ≥ 12.5 cm, a surface estimate ≥ 98.1 cm2, and a flap circumference to skull hemi-circumference ratio ≥ 57.7% are cutoff values that are predictive of survival. The corresponding thresholds that are predictive of favorable outcomes are 13.4 cm, 107.3 cm2, and 62.4%, respectively (12). In the present case, these values were 13.0 cm, 111.2 cm2, and 58.4%, respectively, which are approximately consistent with these parameters.

Bilateral decompression was considered during the clinical course because the patient had bilateral ASDHs and diffuse cerebral swelling. However, at the initial stage, the hematomas were relatively thin, and immediate bilateral decompressive surgery was considered highly invasive. When CT findings progressed 12 h after injury, the left hemisphere, which was the dominant hemisphere, showed more pronounced swelling with rightward midline shift; therefore, left-sided decompressive craniectomy was performed. After recurrent postoperative ICP elevation, additional contralateral decompression was considered but was not performed because postoperative CT showed progression of cerebral contusions and low-density areas, and the patient's family declined further surgical intervention.

The postoperative course also highlights the importance of delayed secondary injury and malignant cerebral edema after decompression. Although decompressive craniectomy and hematoma evacuation substantially reduced ICP, postoperative CT demonstrated expansion of cortical low-density areas. These findings suggest that secondary brain injury had already progressed or continued to evolve despite mechanical decompression. Therefore, ICP normalization does not necessarily indicate reversal of ongoing brain injury. This case emphasizes that continuous ICP monitoring is valuable, but ICP values should be interpreted together with serial CT findings, neurological examination, and the overall clinical course. Nevertheless, the degree of ICP reduction achieved in the current case would likely not have been attained through neurocritical care alone. Given the important contribution of surgical intervention to decreasing ICP, it can be inferred that performing decompressive craniectomy and hematoma evacuation as promptly as possible following burr hole placement represents a crucial step toward improving patient outcomes. Furthermore, the ICP sensor is a critical tool that enables the quantification of ICP, which is otherwise difficult to assess objectively. It has been suggested that performing craniotomy based on continuously monitored ICP values contributes to improving patient outcomes (13). Although further discussion is needed regarding whether an even earlier surgical intervention in our case might have led to a better prognosis, we have reported the quantitative changes in ICP at each surgical step as a valuable clinical observation that may contribute to the advancement of neurocritical care and emergency medicine.

## Conclusion

The present case illustrates the stage-specific decompressive effect of perforation, craniotomy, dural incision, and hematoma removal to ICP reduction in ASDH. However, substantial ICP reduction did not prevent fatal neurological deterioration, indicating that ICP normalization does not necessarily equate to reversal of secondary brain injury. These quantitative data provide valuable insights into the decompressive effect of each surgical step.

## Data Availability

The original contributions presented in the study are included in the article; further inquiries can be directed to the corresponding author.
